# Impact of national partnership to improve dementia care on antipsychotic use duration for dementia residents in nursing homes

**DOI:** 10.1093/gerona/glag060

**Published:** 2026-02-27

**Authors:** Theresa I Shireman, Corinne Roma, Andrew R Zullo, Antoinette B Coe, Lori A Daiello, Christopher J Liu, Derrick Lo, Julie P W Bynum, Lauren B Gerlach

**Affiliations:** Center for Gerontology and Healthcare Research, Brown University School of Public Health, Providence, Rhode Island, United States; Department of Health Services, Policy, and Practice, Brown University School of Public Health, Providence, Rhode Island, United States; Department of Epidemiology, Brown University School of Public Health, Providence, Rhode Island, United States; Center for Gerontology and Healthcare Research, Brown University School of Public Health, Providence, Rhode Island, United States; Center for Gerontology and Healthcare Research, Brown University School of Public Health, Providence, Rhode Island, United States; Department of Health Services, Policy, and Practice, Brown University School of Public Health, Providence, Rhode Island, United States; Department of Epidemiology, Brown University School of Public Health, Providence, Rhode Island, United States; Center of Innovation in Long-Term Services and Supports, Providence Veterans Affairs Medical Center, Providence, Rhode Island, United States; Institute for Healthcare Policy and Innovation, University of Michigan, Ann Arbor, Michigan, United States; Department of Clinical Pharmacy, College of Pharmacy, University of Michigan, Ann Arbor, Michigan, United States; Center for Gerontology and Healthcare Research, Brown University School of Public Health, Providence, Rhode Island, United States; Department of Health Services, Policy, and Practice, Brown University School of Public Health, Providence, Rhode Island, United States; Department of Neurology, Warren Alpert Medical School, Brown University, Providence, Rhode Island, United States; Center for Gerontology and Healthcare Research, Brown University School of Public Health, Providence, Rhode Island, United States; Center for Gerontology and Healthcare Research, Brown University School of Public Health, Providence, Rhode Island, United States; Institute for Healthcare Policy and Innovation, University of Michigan, Ann Arbor, Michigan, United States; Department of Internal Medicine, University of Michigan, Ann Arbor, Michigan, United States; Institute for Healthcare Policy and Innovation, University of Michigan, Ann Arbor, Michigan, United States; Department of Psychiatry, University of Michigan, Ann Arbor, Michigan, United States; (Medical Sciences Section)

**Keywords:** Antipsychotics, Nursing homes, Dementia, Health policy

## Abstract

**Background:**

Federal policies have successfully targeted the prevalence of antipsychotic (AP) exposure in NHs, but the duration of treatment among nursing home (NH) residents has not been reported. We evaluated AP duration and discontinuation within six months in residents with dementia.

**Methods:**

Retrospective cohort study of long-stay NH residents with dementia who newly initiated an AP medication. We evaluated changes in AP duration and discontinuation within six months relative to two federal initiatives: National Partnership to Improve Dementia Care (2012) and inclusion of AP use measures in the NH Star Ratings (2015). We accounted for resident and facility characteristics in a competing-risks analysis establishing the relationship between the two policy periods and AP outcomes.

**Results:**

There were 43 668 new episodes of AP initiation among 38 275 residents. The duration of treatment within six months declined from 125.9 days in the pre-Partnership period to 120.5 days and 120.6 days in the post-Partnership and post-Five Star periods, respectively. Those who initiated APs after the Partnership [adjusted hazard ratio (aHR) = 1.17; confidence interval, 1.10-1.24] and after the Five Star Rating change (aHR = 1.19; 95% CI, 1.07-1.32) policy periods were more likely to stop the medication within 6 months as compared to those who initiated during the prior period.

**Conclusions:**

Federal policies designed to reduce AP prescribing in NH residents with dementia had a nominal impact on treatment duration within the first six months, with more than half continuing treatment beyond 6 months.

## Introduction

People living with dementia (PLWD) frequently experience a range of neuropsychiatric symptoms, such as mood changes, agitation, aggression, wandering, sleep changes, hallucinations, and delusions.^1–4^ Despite limited evidence of clinical benefit and an overt Federal Food & Drug Administration (FDA) Boxed Warning for increased mortality, antipsychotics (APs) are still regularly prescribed to control neuropsychiatric symptoms in PLWD.

To address potentially inappropriate use of APs in nursing home (NH) residents, the Centers for Medicare & Medicaid Services (CMS) created the National Partnership to Improve Dementia Care in 2012 (the Partnership) and subsequently included AP use measures in the NH Star Rating system. CMS reports and several studies have found that the prevalence of AP use in long-stay NH residents has decreased substantially since the start of these initiatives.^5–10^ CMS reported that the national prevalence of antipsychotic use declined from 23.9% in 2011 to 14.5% in 2021, a 39.1% decrease.^8^ In addition to reducing the proportion of residents who receive any AP, it is also important to consider how long they receive treatment. CMS guidance states that when antipsychotic medications are used, NHs should limit exposure through gradual dose reductions and discontinuation as soon as the precipitating symptoms subside.^11^^,^^12^ Given the fluctuating nature of neuropsychiatric symptoms in dementia, reevaluation of the continued need for AP medications is recommended in practice. Given the broader success in reducing the prevalence of AP use in NHs, it is reasonable to assume that the Partnership and changes to the Five Star Rating system may have also decreased the *duration* of AP use among long-stay NH residents. We are not aware of any prior studies that have directly examined the impact of such initiatives on the duration of AP use in a national sample.

NH characteristics and resident-level factors are known to impact antipsychotic exposure through the lenses of resource constraints and structural racism. For instance, for-profit NHs had a higher rate of new exclusionary diagnoses—conditions for which residents are removed from the denominator of antipsychotic medication quality measures—compared to nonprofit facilities.^13^ NHs with a higher proportion of Black residents and residents enrolled in Medicaid have lower quality and more limited resources.^14–16^ Nonpharmacologic management of residents with disruptive behaviors also requires adequate nurse staffing.^14–18^

This study evaluated whether the Partnership and Five Star Rating system changes impacted AP duration and discontinuation within six months of initiation among long-stay NH residents with dementia. In focusing on those residents with dementia, we examined exposure among those for whom the FDA Boxed Warning is specifically targeted. We hypothesized that the duration of AP use would decrease and that discontinuation rates would be higher among these residents who initiated AP in periods after policy changes compared to before, mirroring the trends in reduced overall AP prescribing. Additionally, we hypothesized that patient and facility characteristics—such as percentage of residents with bipolar disorder, facility profit status, percentage of residents on Medicaid, staffing hours per resident day, and proportion of non-Hispanic Black residents—would modify effects given their potential reflection as proxies of differential facility-level resources available to manage behaviors associated with dementia.^14–18^

## Methods

### Study design

We examined the impact of the Partnership and Five Star Rating changes on duration and discontinuation within six months among NH residents with dementia who initiated an AP between 2010 and 2017 using a retrospective cohort design. We examined these outcomes across three policy periods [before the Partnership, between the Partnership (2012) and the Five Star Rating changes (2015), and after the Five Star Rating changes]. We also adjusted our results for proxies of NH resource characteristics: facility profit status, staffing hours, percentage of residents on Medicaid, and percentage of non-Hispanic Black residents.

This work was executed under the terms of a Data Use Agreement with the CMS including a Waiver of Informed Consent and Health Insurance Portability and Accountability Waiver of Authorization for the use of the person-level data.

### Data sources

We created our analytic data file from the Medicare Beneficiary Standard File (MBSF), the Medicare Provider Analysis and Review (MedPAR) claims, fee-for-service Part B claims, and prescription drug event claims (Part D). We also used our internal Residential History File to determine daily beneficiary residence and establish long-stay NH status.^19^ Facility level measures came from the Certification and Survey Provider Enhanced Reporting (CASPER) and the Online Survey Certification and Reporting (OSCAR).

### Study cohort

Our cohort included long-stay NH residents with dementia who started an AP. Long-stay residents were defined as residents who had >100 days in the NH with no more than 10 days outside of a NH. Eligible individuals were diagnosed with dementia based on their first documentation on a claim or on a Minimum Data Set (MDS) assessment; residents were considered to have dementia from that time point forward. We included beneficiaries who were at least 65 years of age at the time of admission to the NH. Furthermore, we restricted the study population to those who initiated a new AP (episode) after at least 90 days without an AP; NH residents could contribute more than one AP episode during the study period. Each episode was treated independently, although 88% of the residents only had a single episode. We did not account for concurrent AP use. Beneficiaries were required to have continuous enrollment in Medicare Parts A, B, and D throughout their observation window. We excluded residents enrolled in Medicare Advantage as their claims were incomplete during the study period, and those with Huntington’s disease, schizophrenia, or a Tourette syndrome diagnosis (so-called exclusionary diagnoses per CMS policy), as they do not contribute to the antipsychotic prevalence measure in the Five-Star NH Ratings.

### Dependent variables

Our outcome variables were AP duration and discontinuation within six months of initiation. Using prescription drug event (Part D) claims, we established daily AP availability. If the days supply for the same medication dispensed on different dates overlapped, we extended the length of the exposure. If a new AP was prescribed, we restarted the days supply. We estimated the duration of AP use in days within 180 days for each policy period using a survival function to account for competing events (censoring and death). We defined AP discontinuation as a period of 30 or more days without drug on hand. For instance, if a resident had 45 days of medication after initiation, stopped it for 31 days, this would be considered a discontinuation event. If they stopped for 29 days but resumed on day 30, they were considered an ongoing user. While we considered a variety of other gap definitions, we settled on 30 days as it is the most common period of refills, even for NH residents and is a measure that has been used in other studies. Follow-up for outcomes began on the date of AP initiation. Outcomes were censored at 180 days post-AP initiation or the earliest date of when the resident was discharged from the NH, disenrolled from fee-for-service Medicare, or disenrolled from Part D.

### Independent variables

For resident characteristics, we incorporated measures from the MDS and from hospital and medical claims that captured changes in temporal trends for NH admissions and clinical conditions potentially linked to fluctuations in mental health (see below). We analyzed the demographics and clinical characteristics of NH residents based on the year of their long-stay admission and used the MDS assessment closest to but preceding July 1 of each year as the reference date. Variables examined were age, sex, race, dual-eligibility status, geographic location (Northeast, South, Midwest, West), activities of daily living (ADL) assessed on a scale from 0 to 28 (where 0 indicates complete independence), incidents of physical and verbal aggression, as well as the presence of anxiety, depression, bipolar disorder, and a composite measure of disease burden known as the Combined Comorbidity Score.^20^ Only resident characteristics that changed considerably over time were included in the competing risk model: these included age, the Combined Comorbidity Score, anxiety, bipolar disorder, and physical aggression.

Facility characteristics were recorded annually on July 1 in CASPER or OSCAR. Facility ownership was categorized as nonprofit NHs or for-profit NHs (for-profit NHs were the reference group). The percentage of residents enrolled in Medicaid was divided into two groups: less than or equal to 62.5% or greater than 62.5%.^16^ The proportion of non-Hispanic Black residents in each NH was classified into three distinct levels: less than 5% (low, reference group), 5% to 15% (intermediate), and over 15% (high). We summed staffing hours per resident day across registered nurses, licensed practical nurses, and certified nursing assistants and categorized as <3 h/day, 3-4.1 h/day, or > 4.1 h/day.^21^

### Analytic approach

First, we generated descriptive statistics of NH resident demographics and clinical characteristics and NH facility characteristics by year from 2010 to 2017 for those who initiated an AP episode. Episodes were categorized according to the time period during which they were initiated: (1) prior to the 2012 CMS Partnership (up to March 2012); (2) when the Partnership was implemented (April 2012-2014), and (3) after the Five Star Quality Rating System went into effect (January 2015). The cause-specific competing-risks were censoring (discharge from NH, disenrollment from fee-for-service Medicare, disenrollment from Medicare Part D or remaining on AP after 180 days), the event of interest (AP discontinuation), or death.

We computed average duration (days) for each AP episode after accounting for competing risks and summarized the means within each policy period. Next, we used a cause-specific competing-risks hazard model to estimate the effect of Partnership and Five Star ratings on AP discontinuation, accounting for facility and selected resident characteristics. We adjusted for resident characteristics and NH resource characteristics. We tested interactions between time period and facility level characteristics.

### Sensitivity analyses

Although APs have FDA approval for the treatment of bipolar disorder, residents with this diagnosis are not excluded from CMS’s NH quality measure and still contribute to a facility’s reported antipsychotic prescribing rate. To explore whether prescribing patterns differ for this clinically indicated use, we modeled AP discontinuation separately for residents with and without bipolar disorder.

Analyses were completed using SAS 9.4 (SAS Institute, Cary, NC). A significance level (alpha) of 0.05 was set using two-sided tests. The institutional review board approved this study.

## Results

### Study cohort and nursing facility characteristics

The number of NH residents with dementia initiating APs and the number of facilities in the sample declined after 2012 (Table 1; details on the results of cohort selection are included in Table S1). On average, residents were in their mid-80s, predominantly non-Hispanic White, about equally dual and nondual enrolled, and mostly resided in the South. Overall, the Combined Comorbidity Score was highest in the latter two years of cohort entry, with there was also a higher prevalence of bipolar disorder and anxiety over time. Physical and verbal aggression rates also increased slightly. ADL scores were comparable year to year.

**Table 1:** Study population characteristics: long-stay nursing home residents with dementia who started an antipsychotic, 2011-2017.

Resident characteristic	Year antipsychotic episode started							
	2010	2011	2012	2013	2014	2015	2016	2017
**Total *N* of unique residents**	5663	6241	6237	6033	5748	4911	4588	3994
**Age, mean (SD), years**	84.5 (7.2)	84.7 (7.3)	85.0 (7.3)	85.4 (7.3)	85.2 (7.4)	85.3 (7.5)	85.3 (7.6)	85.4 (7.6)
**Female, *N* (%)**	4128 (72.9)	4584 (73.4)	4611 (73.9)	4494 (74.5)	4271 (74.3)	3603 (73.4)	3329 (72.6)	2838 (71.1)
**Race/ethnicity, *N* (%)**								
Non-Hispanic White	4684 (82.7)	5194 (83.2)	5118 (82.1)	5005 (83.0)	4753 (82.7)	4116 (83.8)	3890 (84.8)	3361 (84.2)
Non-Hispanic Black	578 (10.2)	657 (10.5)	647 (10.4)	592 (9.8)	560 (9.7)	421 (8.6)	390 (8.5)	353 (8.8)
Hispanic	280 (4.9)	283 (4.5)	345 (5.5)	325 (5.4)	324 (5.6)	278 (5.7)	220 (4.8)	208 (5.2)
Other/missing	121 (2.1)	107 (1.7)	127 (2.0)	111 (1.8)	111 (1.9)	96 (2.0)	88 (1.9)	72 (1.8)
**Full dual-eligible, *N* (%)**	3120 (55.1)	3146 (50.4)	3259 (52.3)	3118 (51.7)	2919 (50.8)	2194 (44.7)	2246 (49.0)	1924 (48.2)
**Geographic residence, *N* (%)**								
Northeast	1165 (20.6)	1338 (21.44)	1275 (20.4)	1233 (20.44)	1116 (19.4)	960 (19.5)	930 (20.3)	813 (20.4)
South	2560 (45.2)	2781 (44.6)	2768 (44.4)	2670 (44.3)	2570 (44.7)	2260 (46.0)	2019 (44.0)	1732 (43.4)
Midwest	1440 (25.4)	1567 (25.1)	1659 (26.6)	1600 (26.5)	1615 (28.1)	1298 (26.4)	1263 (27.5)	1081 (27.1)
West	498 (8.8)	555 (8.9)	535 (8.6)	529 (8.8)	446 (7.8)	393 (8.0)	375 (8.2)	368 (9.2)
**Combined comorbidity score, mean (SD)**	5.3 (3.3)	5.2 (3.4)	5.4 (3.4)	5.2 (3.4)	5.2 (3.3)	5.2 (3.4)	6.1 (3.4)	6.4 (3.4)
**Anxiety, *N* (%)**	3799 (67.1)	4578 (73.4)	4752 (76.2)	4776 (79.2)	4727 (82.2)	4157 (84.6)	3892 (84.8)	3412 (85.4)
**Depression, *N* (%)**	4971 (87.8)	5646 (90.5)	5681 (91.1)	5515 (91.4)	5305 (92.3)	4482 (91.3)	4155 (90.6)	3627 (90.8)
**Bipolar disorder, *N* (%)**	733 (12.9)	834 (13.4)	873 (14.0)	931 (15.4)	968 (16.8)	840 (17.1)	805 (17.5)	663 (16.6)
**Physical aggression, *N* (%)**	1508 (26.6)	1852 (29.7)	1836 (29.4)	1946 (32.3)	1781 (31.0)	1592 (32.4)	1442 (31.4)	1252 (31.3)
**Verbal aggression, *N* (%)**	1947 (34.4)	2293 (36.7)	2403 (38.5)	2467 (40.9)	2284 (39.7)	1954 (39.8)	1882 (41.0)	1581 (39.6)
**ADL score, mean (SD)**	16.9 (7.0)	17.3 (6.7)	17.8 (6.3)	17.7 (6.2)	17.6 (6.1)	17.8 (5.9)	17.7 (5.6)	17.8 (5.6)

Abbreviation: ADL, activities of daily living.

The majority of NHs contributed only one resident in the cohort (Table 2). Aside from slightly lower percentage in 2010, the percentage of residents in the facilities who were Black was consistent, as was the percentage of nonprofit facilities and the percentage of residents on Medicaid. Nursing staff hours remained consistent over time.

**Table 2:** Characteristics of nursing homes that had at least 1 resident who initiated an antipsychotic, 2011-2017.

Nursing home characteristic	Year antipsychotic episode initiated							
	2010	2011	2012	2013	2014	2015	2016	2017
**Number of nursing homes**	4211	4608	4645	4507	4312	3832	3564	3182
**Distribution of residents within nursing homes, *N* (%)**								
1 resident	3147 (74.7)	3416 (74.1)	3439 (74.0)	3379 (75.0)	3264 (75.7)	2999 (78.3)	2808 (78.8)	2558 (80.4)
2 residents	825 (19.6)	871 (18.9)	913 (19.7)	835 (18.5)	802 (18.6)	653 (17.0)	591 (16.6)	515 (16.2)
3+ residents	239 (5.7)	321 (7.0)	293 (6.3)	293 (6.5)	246 (5.7)	180 (4.7)	165 (4.6)	109 (3.4)
**Percentage of residents identified as Black, mean (SD)**	9.8 (18.5)	11.8 (18.1)	11.9 (17.8)	11.9 (17.9)	12.0 (18.0)	11.5 (17.2)	11.4 (17.0)	11.6 (17.2)
**Nonprofit or government-owned, *N* (%)**	1146 (27.2)	1284 (27.9)	1301 (28.0)	1323 (29.4)	1217 (28.2)	1148 (30.0)	1086 (30.5)	937 (29.4)
**Urban location, *N* (%)**	2969 (70.5)	3254 (70.6)	3280 (70.6)	3130 (69.4)	2995 (69.5)	2646 (69.1)	2420 (67.9)	2160 (67.9)
**Percentage of residents on Medicaid, mean (SD)**	63.3 (17.0)	63.0 (18.0)	63.4 (17.5)	62.7 (17.9)	62.4 (17.8)	61.7 (18.2)	61.8 (18.8)	61.0 (19.4)
**Staffing hours per day, mean (SD)**	3.5 (1.1)	3.6 (1.1)	3.5 (1.0)	3.6 (1.1)	3.6 (0.8)	3.6 (0.8)	3.6 (0.8)	3.6 (0.8)
**Distribution of staffing hours/day, *N* (%)**								
<3 h/day	1040 (24.7)	965 (20.9)	1037 (22.3)	869 (19.3)	791 (18.3)	691 (18.0)	719 (20.2)	644 (20.2)
3-4.1 h/day	2496 (59.3)	2839 (61.6)	2821 (60.7)	2796 (62.0)	2685 (62.3)	2367 (61.8)	2091 (58.7)	1907 (59.9)
>4.1 h/day	675 (16.0)	804 (17.4)	787 (16.9)	842 (18.7)	836 (19.4)	774 (20.2)	754 (21.2)	631 (19.8)
**Number of beds, mean (SD)**	132.6 (73.6)	131.2 (71.5)	131.1 (70.8)	130.0 (73.6)	129.0 (71.4)	130.8 (72.1)	128.0 (70.9)	127.9 (70.3)

### AP duration within each policy period

Across the study period, there were 43 668 new episodes of AP initiation among 38 275 unique residents. Most residents, 33 738 or 88.2% of the study population, had a single episode. There was 3848 residents (10.1%) who had two episodes and 689 (1.8%) had three or more episodes. Most episodes occurred in NHs with a low percentage of Black residents, a majority of residents on Medicaid, and under for-profit ownership (Table S2).

**Figure 1:**
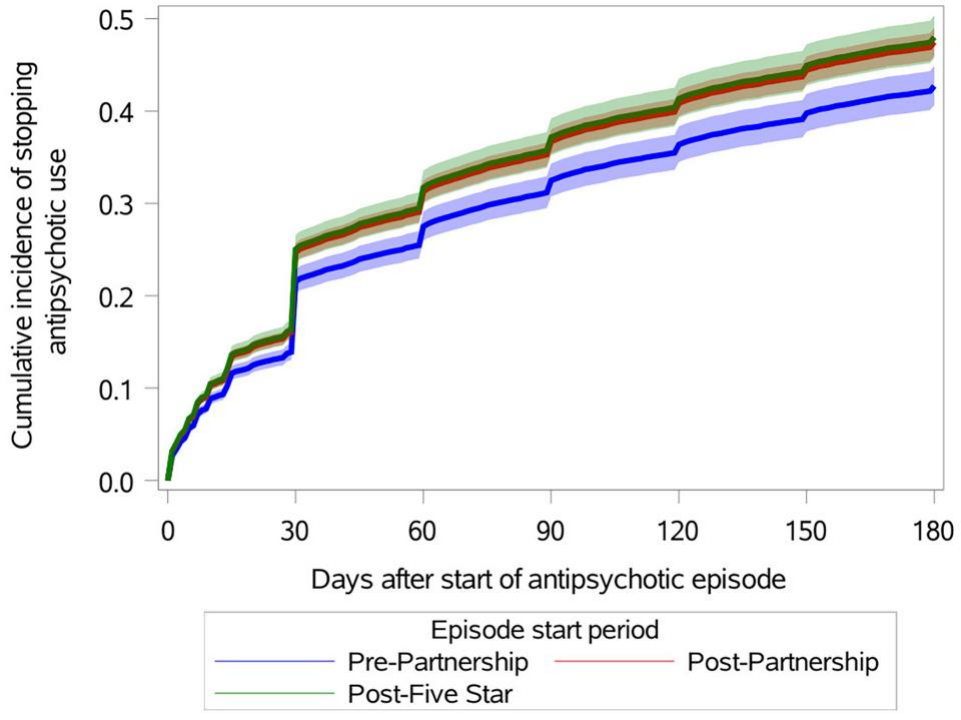
Proportion of nursing home residents with dementia who discontinued a new antipsychotic prescription within 6 months across 3 federal policy periods, 2011-2017. Figure displays cumulative incidence of 6-month antipsychotic discontinuation following initiation, stratified by policy period: pre-Partnership (blue), post-Partnership (red), and post-Five Star (green). Estimates are derived from fully adjusted competing risks models accounting for the competing risk of death. Models adjust for resident-level characteristics (age, comorbidity, anxiety, bipolar depression, and physical aggression) and facility-level characteristics (ownership, percent of residents that were non-Hispanic Blacks, staffing hours, percent of residents on Medicaid). Stepwise shape reflects the common dispensing of 30-day medication supplies.

Within the 6-month follow-up, the average time to discontinue AP use within each policy period, after accounting for competing risks, declined from 125.9 days in the pre-Partnership period to 120.5 days and 120.6 days in the post-Partnership and post-Five Star periods, respectively. In Figure 1, we plotted the cumulative incidence of AP discontinuation. During the pre-Partnership period (blue line), the cumulative AP discontinuation rate was 0.4, meaning that 40% of the cohort had discontinued their AP within six months. In the subsequent two periods, discontinuation increased (red and green lines), although their confidence bands (shaded areas) overlapped, indicating they were not significantly different. Those who initiated during the post-Partnership (red line) and post-Five Star (green line) periods experienced discontinuation rates closer to 0.5. In other words, more than 50% of NH residents who initiated an AP were estimated to still be taking the medication after six months regardless of the policy period.

### AP discontinuation within 6 months

NH residents with dementia who initiated an AP in the post-Partnership [adjusted hazard ratio, (aHR) = 1.17; 95% confidence interval (CI), 1.10-1.24] and post-Five Star Rating (aHR = 1.19; 95% CI, 1.07-1.32) policy periods were more likely to stop the medication within 6 months as compared to those who initiated during the pre-Partnership period (Table 3). Other factors positively associated with AP discontinuation included increasing resident age and higher Combined Comorbidity Scores and higher percentages of NH residents who were Black (Table 3). Presence of a co-morbid mental health condition, such as anxiety (aHR = 0.86; 95% CI, 0.83-0.89), bipolar disorder (aHR = 0.86; 95% CI, 0.82-0.89), or depression (aHR = 0.88; 95% CI, 0.84-0.93) was associated with lower 6-month AP discontinuation.

**Table 3:** Factors associated with AP discontinuation within 180 days, 2011-2017.

Analysis of maximum likelihood estimates							
Parameter	Parameter estimate	Standard error	Chi-square	Pr > ChiSq	Hazard ratio	95% HR confidence limits	
**Post-partnership**	0.1564	0.02917	28.7503	<.0001	1.17	1.10	1.24
**Post-five star**	0.1730	0.05293	10.6819	.0011	1.19	1.07	1.32
**Quarters after first**	0.00773	0.00235	10.7682	.001	1.01	1.00	1.01
**Resident characteristics**							
**Age**	0.01053	0.00103	104.2583	<.0001	1.01	1.019	1.013
**Combined comorbidity score**	0.01215	0.00223	29.6572	<.0001	1.01	1.008	1.017
**Anxiety**	−0.15231	0.01807	71.0218	<.0001	0.86	0.83	0.89
**Bipolar disorder**	−0.15607	0.02142	53.0735	<.0001	0.86	0.82	0.89
**Depression**	−0.12375	0.02519	24.1368	<.0001	0.88	0.84	0.93
**Physical aggression**	−0.02759	0.01599	2.9776	.0844	0.97	0.94	1.004
**Nursing home characteristics^a^**							
**Intermediate percent Black residents (5%-15%)**	0.0515	0.01917	7.2192	.0072	1.05	1.01	1.09
**Higher percent Black residents (15% or more)**	0.09606	0.01834	27.4395	<.0001	1.10	1.06	1.14
**Higher percent Medicaid (>62.5%)**	−0.04938	0.01548	10.1816	.0014	0.95	0.92	0.98
**Nonprofit or government-owned**	−0.03334	0.01682	3.9316	.0474	0.97	0.94	1.00
**Staffing time, 3-4.1 h/day**	0.00788	0.01881	0.1755	.6753	1.01	0.97	1.05
**Staffing time, >4.1 h/day**	−0.03412	0.02416	1.9952	.1578	0.97	0.92	1.01

a Referent groups: low percent Black residents, <5%; lower percent Medicaid, ≤62.5%; for-profit ownership; lower staff time, <3 h/day.

Abbreviations: AP, antipsychotic; HR, hazard ratio.

NHs with an intermediate (aHR = 1.05; 95% CI, 1.01-1.09) or higher (aHR = 1.10; 95% CI, 1.06-1.14) percentage of residents who were Black were more likely to discontinue the AP within 6 months. In contrast, NHs with a higher percentage of residents on Medicaid (aHR = 0.95; 95% CI, 0.92-0.98) were less likely to discontinue APs within 180 days. Nonprofit NH status (aHR = 0.97; 95% CI, 0.94-1.00) and staffing hours per resident day were not significantly associated with discontinuation (3-4.1 h/day: aHR = 1.008; 95% CI, 0.97-1.05; >4.1 h/day, aHR = 0.97; 95% CI, 0.92-1.01). Interactions between NH characteristics and policy periods were not significant.

### Sensitivity analysis

When the cohort was limited to those with bipolar disorder, neither policy period was associated with AP discontinuation (Table S3). Factors associate with AP discontinuation in this subgroup were age (aHR = 1.03; 95% CI, 1.01-1.02), anxiety (aHR = 0.81; 95% CI, 0.72-0.90), residing in a NH with a higher percentage of Black residents (aHR = 1.11; 95% CI, 1.01-1.22), in a NH with a higher percentage of Medicaid residents (aHR = 0.88; 95% CI, 0.81-0.95), or higher staffing hours (aHR = 0.86; 95% CI, 0.83-0.89).

## Discussion

This study quantified AP treatment duration and likelihood of AP discontinuation within six-months of initiation among long-stay NH residents with dementia following federal initiatives aimed at reducing AP use. In total, the average number of days to AP discontinuation within the six-month follow-up dropped by roughly 5 days: the average resident received an AP for 120-125 out of 180 days. Over 50% of NH residents with dementia who initiated an AP remained on the medication for 6 months or longer. We also examined whether the results differed across NH characteristics across three policy periods. Consistent with our hypothesis, AP initiators were more likely to discontinue the AP (within 6 months) after the 2012 Partnership in comparison to the period prior to the Partnership. The 2015 Five Star Rating system modification did not further alter the discontinuation rate significantly, however.

Residents with mental health conditions, particularly bipolar disorder, depression, and anxiety and those in NHs with high Medicaid coverage and nonprofit facilities were less likely to experience discontinuation within six months. Residents in NHs with a higher percentage of Black residents were more likely to discontinue APs. Staffing hours were not significantly associated with discontinuation. While previous studies have demonstrated reductions in AP prescribing following these federal initiatives, findings offer novel insights into the *duration* of antipsychotic use and prescribing behaviors among NH residents with dementia, an area with limited study.

The reduction in average time to AP discontinuation following the 2012 Partnership and through the Five Star rating change suggests that these policies have had some influence on prescribing behaviors. Our findings align with CMS data that report a decrease in prevalence of AP use and recent findings that report a decrease in duration of potentially harmful medication use in the post-policy periods.^6^ However, the small magnitude of the reduction in exposure duration occurring within six months of AP treatment suggests that the clinical impact may be modest at best, considering that even relatively brief periods of use have been linked with considerable harms.^22–24^ Nearly two decades of research have demonstrated that both short- and long-term AP treatment in dementia populations is associated with multiple risks, including acute adverse consequences such as excessive sedation, fractures, head injury, and stroke, as well as long-term impacts, e.g., Parkinsonism, cognitive and functional decline, and increased mortality.^25^ Nonetheless, persistent use of APs beyond 6 months in more than half of NH residents with Alzheimer’s disease and related dementias (ADRD) raises concerns about efforts in NHs to follow guidelines which recommend attempts at gradual dose reduction and discontinuation of APs if possible.

Increased likelihood of persistent AP use in residents with dementia and comorbid mental health conditions highlights the complexity of clinical decision making with AP prescription.^26^ Short-term AP use may be necessary and appropriate for some NH residents to manage neuropsychiatric symptoms associated with dementia, and ongoing use may certainly be appropriate for those with indicated mental health disorders. Risks and benefits associated with AP use should be continuously re-evaluated as the resident’s condition and symptoms change, and monthly drug regimen reviews are one avenue to identify appropriate duration of AP use and to initiate tapering in patients who no longer require APs. Federal regulations require pharmacists to evaluate AP medication orders for appropriate clinical indications when prescribed on a scheduled regimen or as needed for more than 14 days. Likewise, quarterly Minimum Data Set assessments require nurses to determine whether residents continue to exhibit behaviors warranting the use of APs and an appropriate indication for treatment. However, policy alone is unlikely to lead to AP deprescribing in NHs. Describing AP in NHs requires addressing nursing staff who may have positive attitudes toward AP effectiveness and lack awareness of the negative effects of APs.^27^ Proper use of team-based assessments can lead to AP discontinuation in small scale studies,^28^^,^^29^ but insufficient resources remain a barrier to implement nonpharmacologic interventions or effective medication reviews.^27^^,^^28^^,^^30^ In addition, the US Department of Health and Human Services Office of the Inspector General is currently examining surveyor reports to establish the relationship between citations and antipsychotic use following concerns about falsification of schizophrenia diagnoses.^31^^,^^32^

NHs with higher Medicaid coverage were less likely to discontinue AP use. This is potentially attributed to financial constraints, as these facilities often have lower reimbursement rates compared to facilities with higher proportions of private pay and Medicare residents.^16^^,^^33^^,^^34^ Consequently, these NHs may have limited ability to invest in staff training for managing dementia-related behaviors or resource-intensive nonpharmacological interventions, potentially creating an over-reliance on APs. On the other hand, staffing hours per resident day were not significantly associated with AP discontinuation for the overall cohort, while we did see less discontinuation among residents with bipolar disorder in NHs with higher staffing levels.

Nonprofit facilities were less likely to discontinue AP use compared to for-profit facilities. One potential explanation is potential floor effects in not-for-profit NHs which typically have lower use of APs compared to for-profit NHs,^35–38^ leaving less room for dose reductions. Another hypothesis is that for-profit NHs more heavily relying on APs to manage behaviors, rather than hiring additional nursing staff, providing continuing education on managing aggression, or utilizing other nonpharmacological strategies which would be more costly to the NH than drugs covered by insurance.^35^

Interestingly, facilities with a higher percentage of Black residents were more likely to discontinue AP use within six months of initiation. Although additional exploration of this finding is beyond the scope of this research, the results of previous studies of disparities in NH utilization and quality of care among PLWD may offer some insights. In general, Black and Hispanic individuals with dementia are more likely to reside in segregated, lower quality NH with fewer resources (e.g., dementia special care units) compared with White residents.^39^ Furthermore, notable differences in dementia stage at NH admission may be an integral determinant of AP treatment duration. Current evidence suggests that relative to White residents, Black residents have more severe cognitive and functional impairments when entering NH care and Black and Hispanic residents with dementia have less severe behavioral symptoms than their White peers.^39–43^ It is possible that cultural beliefs on the part of patients, their caregivers, and staff may influence AP initiation and discontinuation, as well as variable consent practices and systematic racism across facilities. Such factors associated with NH care disparities may have also impacted greater AP discontinuation among Black residents.

This study has several important limitations. First, our study is limited to long-term stay NH residents with dementia enrolled in fee-for-service Medicare and may not generalize to short-term stay residents or the growing population of residents enrolled in Medicare Advantage or commercial health plans. Second, we limited our follow-up window to six months. We were unable to identify existing literature quantifying AP duration to set a standard, and we had to balance follow-up time with power considerations and data constraints. We opted for six months as it represents two cycles of MDS reviews and six months of pharmacists’ reviews. While claims do not indicate if the fill was for a PRN vs scheduled medication, pharmacies are required to refund medications returned from the NHs and/or only provide PRN medications when the NH needs to be restocked at the resident-level. Given this practice, the impact of PRN use would be limited and the daily array of medication availability should be a strong reflection of daily administration. If a PRN medication is being used daily, then the claims daily count would reflect ongoing administration. Given the observational nature of this study, there is also the possibility of residual confounding. Additionally, our study does not account for medication dosing, so we may not have captured gradual dose reductions. Facilities may have attempted gradual dose reductions with reemergence of clinical symptoms warranting continued AP treatment. Prescription claims may be an imprecise measure of actual consumption, although since nursing staff administer medications in NHs, the potential for bias is likely nominal. In addition, our focus on NHs does not directly account for families’ and/or caregivers’ perspectives and decisions on the need for antipsychotics. Lastly, the policy interventions we studied may seem dated, however, most of the decline in antipsychotic use had occurred by 2017, the end of our study period, at which point the prevalence of AP use was 15.1% versus 14.5% in 2021.

## Conclusion

We found that both the 2012 Partnership and the subsequent 2015 Five Star Rating periods were associated with a modest, yet statistically significant, increase in the likelihood of AP discontinuation within six months. However, the average time to discontinuation was reduced by only about five days and over 50% of residents remained on therapy beyond six months. These findings suggest that while federal policies have spurred some positive change, their impact on reducing prolonged AP exposure is limited. A comprehensive approach that incorporates enhanced clinical strategies, meaningful engagement with families and NH staff, and policy supports—such as routine medication reviews, increased support for nonpharmacological interventions, and targeted resource allocation—is needed to help minimize medication-related harms for NH residents with dementia moving forward.

## Supplementary Material

glag060_Supplementary_Data

## Data Availability

The data used in this analysis are not publicly available but can be accessed with a data use agreement with the Centers for Medicare and Medicaid Services (CMS). Meta data for this project, in compliance with the NIH Data management and sharing requirements, are available at https://doi.org/10.26300/6bcs-b522.
