# Supplementary material for: Impact of national partnership to improve dementia care on antipsychotic use duration for dementia residents in nursing homes
Source: J Gerontol A Biol Sci Med Sci. 2026 Feb 27;81(4):glag060. doi: 10.1093/gerona/glag060 (PMC13014163; doi:10.1093/gerona/glag060)
Supplement: glag060_Supplementary_Data [file glag060_supplementary_data.pdf]

Impact of National Partnership to Improve Dementia Care on Antipsychotic Use Duration for Dementia Residents in Nursing Homes

Supplemental Material

Corresponding Author

Theresa I. Shireman, PhD

Center for Gerontology and Healthcare Research

Brown University School of Public Health

Room 621, 121 South Main Street, Rhode Island, United States 02903

Tel.: 401-863-3170

Email: Theresa_shireman@brown.edu

Table of contents

Supplemental Table 1. Cohort selection: NH Residents with dementia initiation an antipsychotic medication

Supplemental Table 2. Number of new AP episodes by NH characteristics across policy periods

Supplemental Table 3. Factors associated with AP discontinuation within 180 days for those with bipolar disorder

Supplemental Table 1. Cohort selection: NH Residents with dementia initiation an antipsychotic medication

step	criterion	# people (change from last step)	# person-months (change from last step)
1	LSNH sample from 2010-2017	2,160,033	51,263,635
2	In 20% sample at least one year from 2010-2017	524,566 (-1,635,467)	12,605,480 (-38,658,155)
3	65+ years of age on admission date	499,324 (-25,242)	11,832,800 (-772,680)
4	Never Huntington's/Tourette's/schizophrenia dx, 2007-2019	454,367 (-44,957)	10,488,471 (-1,344,329)
5	AP episode starting on or after long-stay date while in NH, 2010-2017	103,205 (-351,162)	1,355,323 (-9,133,148)
6	+ ADRD diagnosis before start of episode	83,994 (-19,211)	1,095,761 (-259,562)
7	+ enrolled in FFS & Part D during 90 days prior to start of episode	76,884 (-7,110)	989,214 (-106,547)
8	+ no AP days' supply in 90 days prior to start of episode	39,238 (-37,646)	365,210 (-624,004)

Supplemental Table 2. Number of new AP episodes by NH characteristics across policy periods, 2011-2017

	**Pre-Partnership**	**Post-Partnership**	**post-Five Star change**
Number of AP episodes	13,581	16,538	13,549
Percent of Black residents in facility			
None up to 5%	7,882 (58.04)	8,521 (51.52)	7,147 (52.75)
5% up to 15%	2,274 (16.74)	3,726 (22.53)	2,996 (22.11)
15% or more	3,425 (25.22)	4,291 (25.95)	3,406 (25.14)
Percent of residents on Medicaid in facility			
Less than or equal to 62.5%	5,507 (40.55)	6,967 (42.13)	6,060 (44.73)
More than 62.5%	8,074 (59.45)	9,571 (57.87)	7,489 (55.27)
NH profit status			
For-profit ownership	9,788 (70.27)	11,729 (70.92)	9,422 (69.54)
Not for-profit ownership	3,793 (27.93)	4,809 (29.08)	4,127 (30.46)
Staffing levels (registered nurses, licensed practical nurses, + certified nurse aides)			
< 3 hours/day	3042 (22.4)	3266 (19.7)	2586 (19.1)
3-4.1 hours/day	8282 (61.0)	10241 (61.9)	8153 (60.2)
>4.1 hours/day	2257 (16.6)	3031 (18.3)	2810 (20.7)

Supplemental Table 3. Factors associated with AP discontinuation within 180 days for those with bipolar disorder, 2011-2017

Maximum Likelihood Estimates							
**Parameter**	**Parameter Estimate**	**Standard Error**	**Chi-Square**	**Pr > ChiSq**	**Hazard Ratio**	**95% HR Confidence Limits**	
Post-Partnership	0.12559	0.07953	2.494	0.1143	1.134	0.97	1.325
Post-Five Star	0.13911	0.14073	0.9772	0.3229	1.149	0.872	1.514
Quarters after first	0.00913	0.0063	2.0957	0.1477	1.009	0.997	1.022
**Resident characteristics**							
Age	0.01319	0.00266	24.5961	<.0001	1.013	1.008	1.019
Combined comorbidity score	0.01098	0.00599	3.3575	0.0669	1.011	0.999	1.023
Anxiety	-0.21758	0.05784	14.1524	0.0002	0.804	0.718	0.901
Depression	-0.01689	0.121	0.0195	0.889	0.983	0.776	1.246
Physical aggression	0.06887	0.04284	2.5843	0.1079	1.071	0.985	1.165
**Facility characteristics**							
Intermediate percent Black residents (5-15%)	0.05065	0.04973	1.0372	0.3085	1.052	0.954	1.16
Higher percent Black residents (15% or more)	0.10555	0.04833	4.7692	0.029	1.111	1.011	1.222
Higher percent Medicaid (>62.5%)	-0.12092	0.04232	8.1621	0.0043	0.886	0.816	0.963
Non-profit or government-owned	-0.00853	0.0463	0.034	0.8538	0.992	0.905	1.086
Staffing time, 3-4.1 hours/day	-0.07104	0.04886	2.1139	0.146	0.931	0.846	1.025
Staffing time, >4.1 hours/day	-0.05003	0.06414	0.6083	0.4354	0.951	0.839	1.079
